# SPEKcheck — fluorescence microscopy spectral visualisation and optimisation: a web application, javascript library, and data resource

**DOI:** 10.12688/wellcomeopenres.14703.1

**Published:** 2018-07-30

**Authors:** Mick A. Phillips, David M. Susano Pinto, Ian M. Dobbie

**Affiliations:** 1 Micron Advanced Bioimaging Unit, Department of Biochemistry, University of Oxford, South Parks Road, Oxford, OX13QU, UK

**Keywords:** Microscopy, Fluorescence, Excitation, Modelling

## Abstract

Advanced fluorescence imaging methods require careful matching of excitation sources, dichroics, emission filters, detectors, and dyes to operate at their best. This complex task is often left to guesswork, preventing optimal dye:filter combinations, particularly for multicolour applications. To overcome this challenge we developed SPEKcheck, a web application to visualise the efficiency of the light path in a fluorescence microscope. The software reports values for the excitation efficiency of a dye, the collection efficiency of the emitted fluorescence, and a "brightness" score, allowing easy comparison between different fluorescent labels. It also displays a spectral plot of various elements in the configuration, enabling users to readily spot potential problems such as low efficiency excitation, emission, or high bleedthrough. It serves as an aid to exploring the performance of different dyes and filter sets.

## Introduction

Modern fluorescence microscopy has been transformed by the availability of custom designed multi-layer dielectric filters and dichroic/polychroic mirrors ^1, 2^, allowing the creation of optimised optical setups for a wide range of excitation sources, dyes, and emission paths. Given the ever-evolving landscape of new imaging modalities ^3– 6^, detailed knowledge of the expected spectral transmission can be especially informative for both custom-built and commercial microscopes.

Computation of the expected spectral transmission is often performed manually in a spreadsheet: the spectrum of each component and dye is pasted into separate columns and combined to calculate the resulting transmission. However, the spectral data for different components may be provided with different sample spacing and ranges, and for filters in transmission or reflection mode. The data therefore requires extensive pre-processing before it can be combined to enable comparison of different setups, resulting in a time-consuming and error-prone process.

We have developed SPEKcheck, a web application to perform these calculations. We provide an online instance of SPEKcheck on our own website, as well the source code for other sites to provide their own instance of SPEKcheck configured for the microscopes they have available.

Other microscope spectrum viewers exist such as the online tools in company websites like Thermo Fisher, Chroma, or Semrock. However, SPEKcheck has a number of additional features:

- The emission spectrum of the dye is combined with those of filters in the emission path, allowing modelling and visualisation of the transmission spectrum. The efficiency of a microscope’s excitation path may be similarly assessed by combining the light source spectrum with those of filters in the excitation path.
- An arbitrary number of dichroic or polychroic mirrors and emission filters can be stacked, with each in either reflection or transmission, to fully model arbitrarily complicated systems.
- Detector spectra can be added to clearly illustrate the difference in detection efficiency between, for instance, the human eye, sCMOS or CCD camera, and a PMT.
- Pre-set configurations can be defined in a configuration file, allowing a lab or facility to model the instruments they have available to their users.
- Adding new dyes, mirrors or filters only involves adding a text file of the dye or filter spectral response to the local installation and updating an index file.
- By including data on dye extinction coefficients and quantum yields SPEKcheck provides reasonable estimates of the relative brightness between different dyes.
- An ‘Optimise dyes’ function can be used to determine the best dyes to use with a given filter configuration and excitation source.
- SPEKcheck is free software enabling facilities to provide an extended and personalised instance to their own users.
- The underlying SPEKcheck library is decoupled from the application and can be used to developed other tools

Together, these features make SPEKcheck a simple yet extremely powerful tool for assessing the excitation and emission efficiency of a fluorescence microscopy setup. Once an existing setup has been defined, it is then easy to model how changes to individual components — dyes, light sources, and optical components — will affect the output signal.

## Methods

### Operation

**Figure 1.**
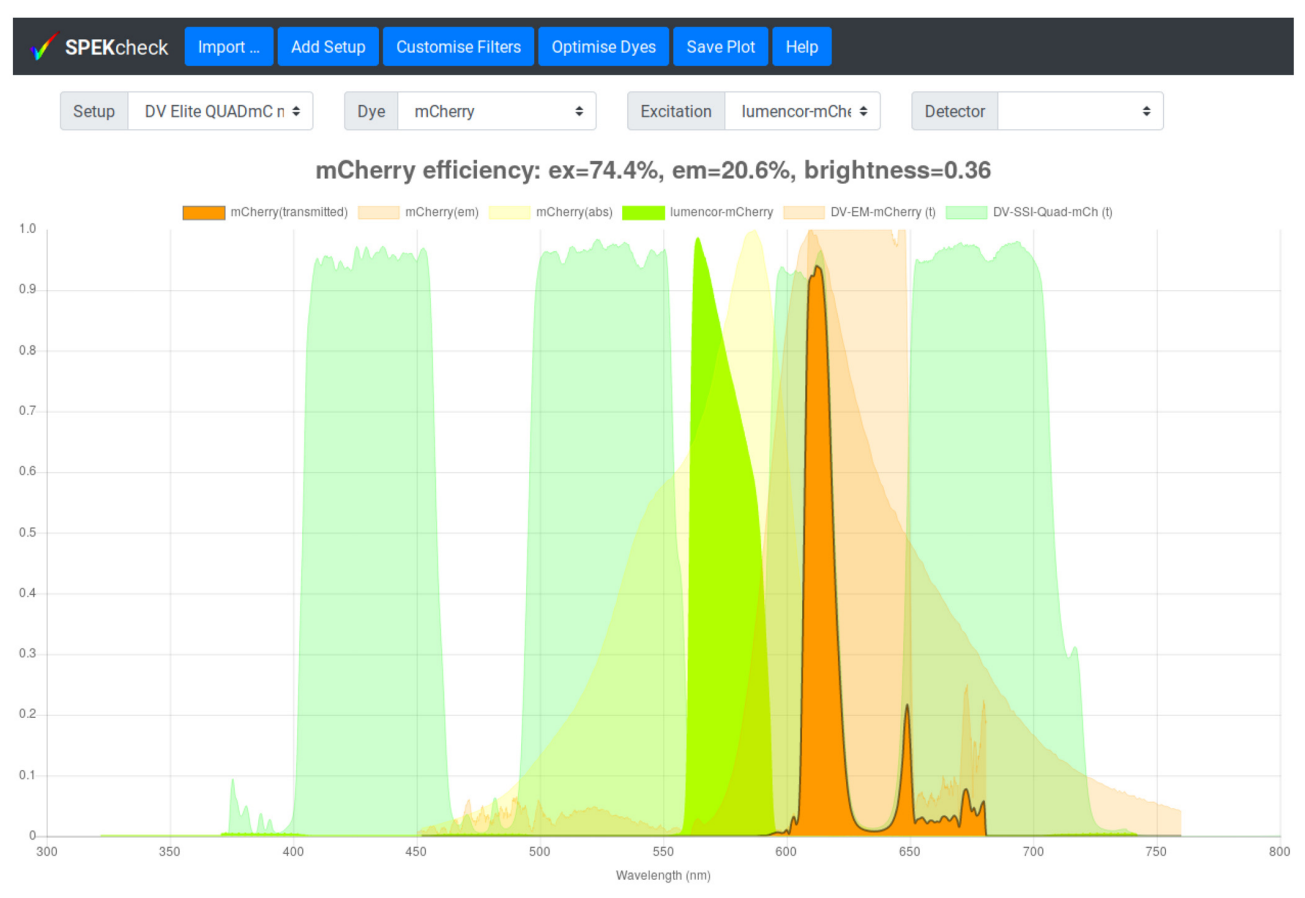
Display of SPEKcheck with a predefined setup for a DV Elite microscope and a mCherry sample. It shows that this setup excites mCherry with an efficiency of 74% and collects 20.6% of the emitted light, leading to a relative brightness of 0.36. See also Figure 2 for a comparison with other dyes. The spectrum for the total transmitted light is shown in bright orange, a dark grey outline, and above all other spectra. The spectrum for the excitation light source is show in bright green, along with the dye absorption and emission spectra in a fainter orange. The emission dichroics and filters are also shown in fainter shades with different tones. Individual traces can be toggled on or off by clicking on the corresponding names in the plot legend.

In the most basic use of SPEKcheck, a predefined microscope configuration, such as ‘DV Elite QUADmC mCherry’, is selected from the ‘Setup’ menu. This will display the excitation and emission efficiency, relative brightness of the fluorescent signal, and the transmission spectra of the different system components and emitted fluorescence ( Figure 1). The displayed components are the excitation light source modulated by the filters in the excitation path, the dye absorption (abs) and emission (em), the dichroic and filters in the emission path, and the detector. The final emitted fluorescence spectrum appears in the brightest colour, with a dark thick outline to emphasise it.

In addition to ‘Setup’, three other selections are available in the basic interface: ‘Excitation’, ‘Dye’, and ‘Detector’. While predefined setups may include a pre-selection for each, these can be changed independently, enabling quick visualisation of their effect in a given setup. If a dye is selected independent from a setup selection, that dye selection will be kept fixed while changing setups.

**Figure 2.**
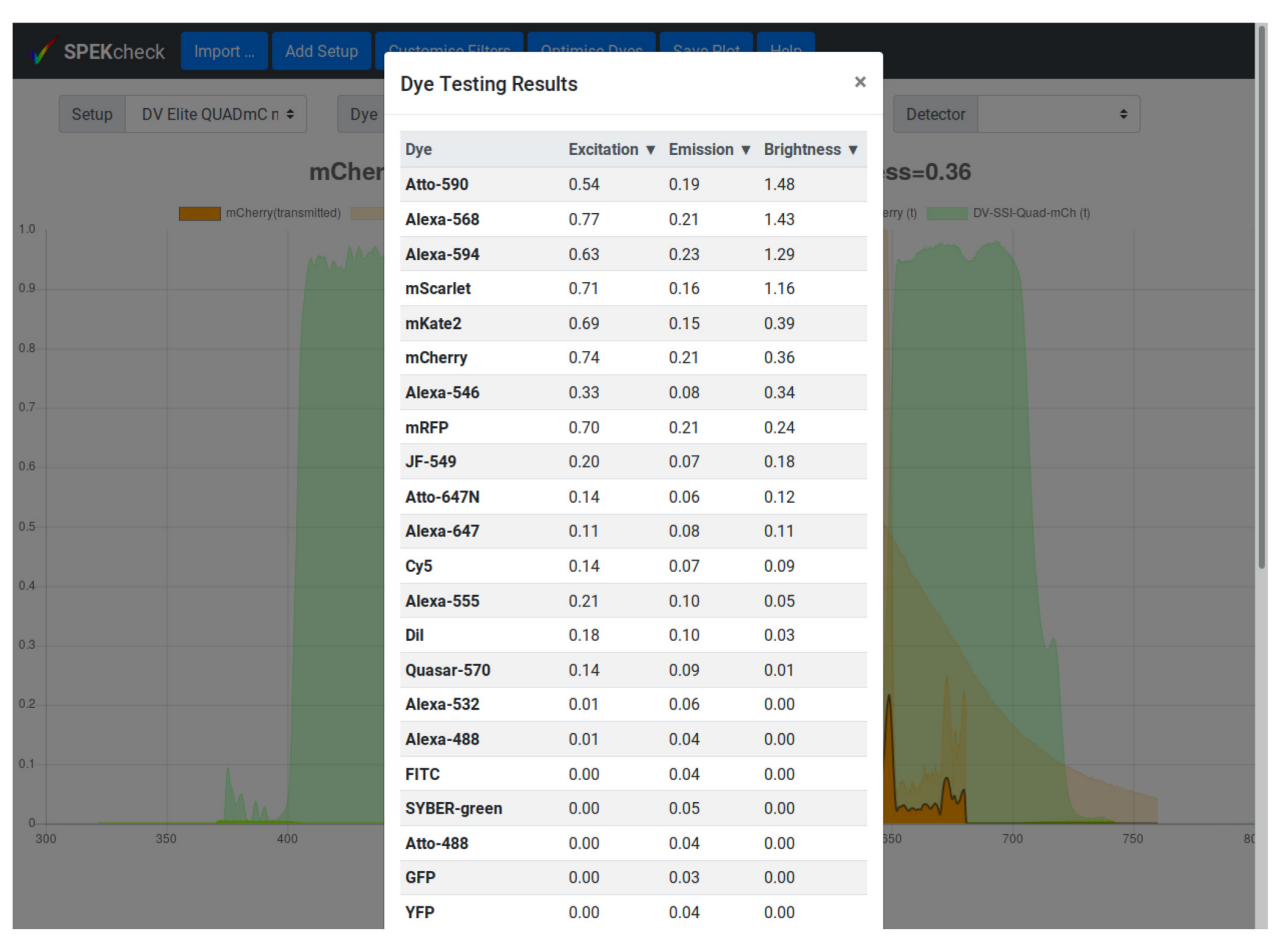
The output of the ‘Optimise Dye’ button from the configuration in Figure 1. By default, the results are sorted by brightness value and Atto-590, can be seen to have the highest brightness at 1.48, followed by Alexa-568, and Alexa-594. The dyes can be ordered by excitation or emission efficiencies by clicking on the column headers.

A further ‘Optimise Dyes’ button tests the entire collection of dyes with all other parameters unchanged and displays a list of their excitation and emission efficiencies, and brightness in tabular form ( Figure 2).

**Figure 3.**
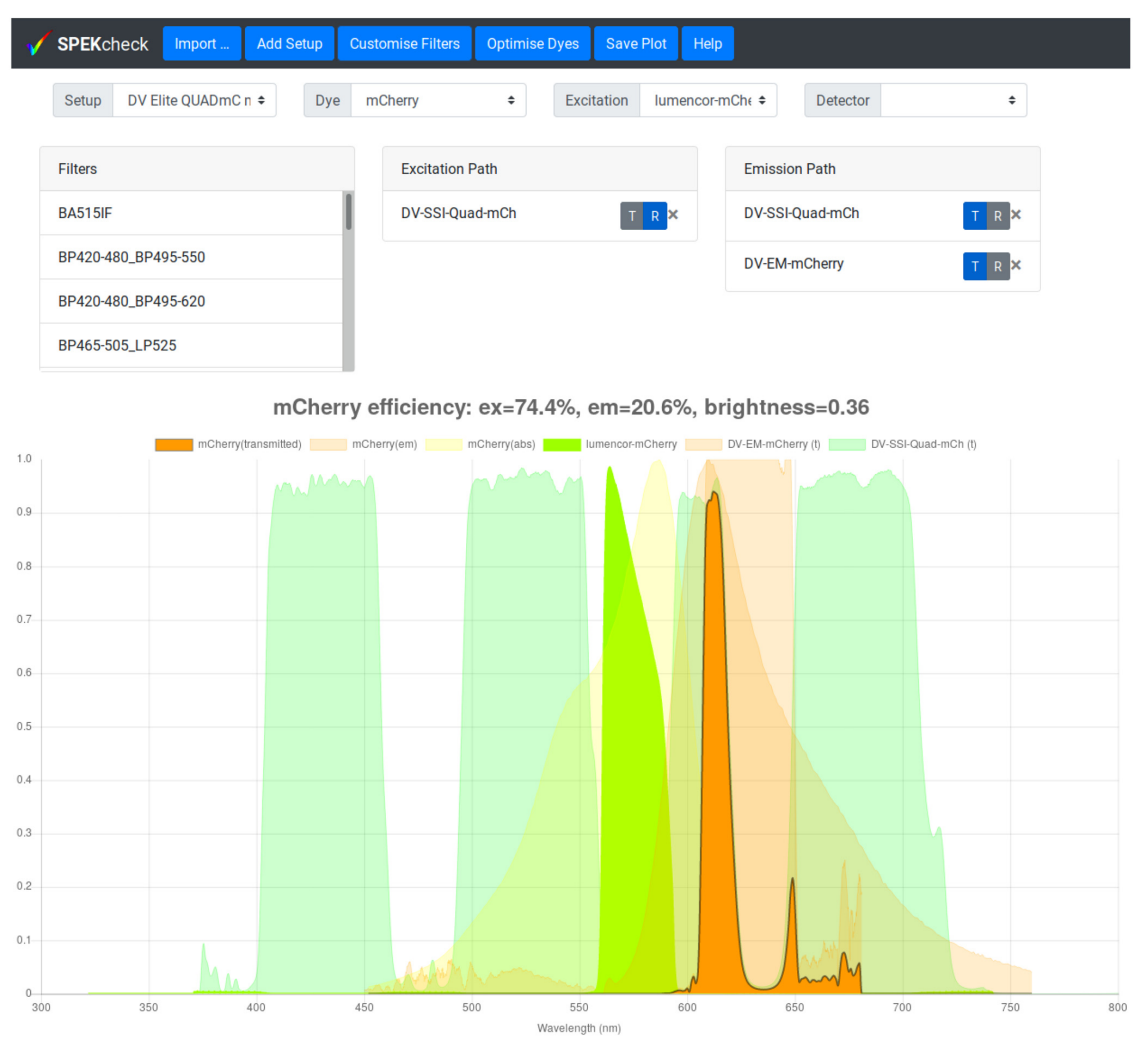
The filter set ‘DV Elite QUADmC mCherry’ has been selected and then ‘Customise Filters’ button pressed to reveal the exact configuration. It can be seen that the excitation path has one dichroic in reflection mode, DV-SSI-Quad-mCh. Because a dichroic is part of both the emission and excitation light paths, it also appears in the emission path in transmission mode, note the blue R button in the Excitation Path and T button in the emission Path. In addition, the emission path includes the DV-EM-mCherry bandpass filter in transmission mode. The transmission or reflection mode of filters and dichroics can be toggled by clicking on the ‘T|R’ slider, or they can be removed from a path by clicking on the X button. New filters or dichroics can be added by dragging from the Filters list on the left into the the relevant Path box.

A more complex interface provides full control over the filters and dichroics in the light path. Clicking on the ‘Customise Filters’ button reveals three additional lists ( Figure 3). The first list, named ‘Filters’, shows all available filters. The two other, named ‘Excitation Path’ and ‘Emission Path’, show the filters currently in their respective paths. Filters can be added to a path by dragging them from the ‘Filters’ list into either path, they can be removed from a path by clicking on their ‘X’ button, and the mode of each filter in a path can be flipped between transmission (T) and reflection (R) through the ‘T|R’ toggle.

### Local configuration

Users can import their own components (i.e. spectra for their own detectors, dyes, excitation sources, and filters) through the ‘Import...’ dialog. Users can also save the currently defined custom setup through the ‘Add Setup’ dialog which saves the current configuration and adds it as a selection at the bottom of the ‘Setup’ menu. However, this configuration is temporary and is not retained between sessions.

To permanently add new components, their data files must be added to the data directory, and their names to the matching json file. To permanently add new setups, they must be defined on the data/setups.json file. To simplify the process of writing a new setup, the ‘Add Setup’ button will also show the required json fragment which can be cut and pasted into the data/setups.json file.

Full documentation for SPEKcheck, including instructions for the different installation types and file formats, is included in the SPEKcheck documentation which is part of the release and online at https://www.micron.ox.ac.uk/software/spekcheck/help.html.

### Control via the URL

The URL fragment and query components can be used to control the initialisation state of SPEKcheck. Adding the string #setup=<name> to the end of the URL will load SPEKcheck with the setup <name>, providing links to specific configurations. The addition of the string ?setup=<name> will filter the setups so that only those containing the string <name> are displayed, providing links for a version of SPEKcheck that only provides a subset of setups for selection.

For example, the fragments #setup=OMXv3 GR Green, #dye=GFP, or #excitation=halogen-lamp will initialise SPEKcheck with these selections. The queries ?setup=OMX or ?setup=DV will filter the setup collection for names containing the relevant text.

### Requirements

SPEKcheck is both a javascript library and a web application. It makes extensive use of HTML5 and ECMAScript 2015 features and thus requires a relatively recent web browser. It should run on any browser that supports these standards. We have tested SPEKcheck on the desktop and on mobile versions of Chrome, Edge, Firefox, Safari, and Silk web browsers. Note that Internet Explorer is *not* supported.

It should be noted that mobile browsers do not provide click-and-drag functionality so the ‘Customise Filters’ controls are not able to create new setup configurations on these browsers. However all other functionality is supported.

The SPEKcheck web application is fully client-side and so does not require a web server. In addition, releases include all external dependencies enabling SPEKcheck to run completely offline.

### Availability

SPEKcheck is free software released under the GNU General Public License (GPL) version 3 or later. Releases are available for download on both GitHub and Zenodo ^7^. The git repository in github is public and includes the source code to prepare new releases in a Makefile.

### Implementation

SPEKcheck is structured after a Model-View-Presenter (MVP) pattern ^8^. In this pattern, Models encapsulate the data and problem logic, Views are representations of data, and a Presenter handles user input redirecting it to the Models or Views. At the core of SPEKcheck is an instance of the Setup and SetupPlot classes. The Setup is our Model of an optical setup, and SetupPlot is the View of its multiple spectra, which listens to change events from Setup. User interactions on the GUI modify the Setup instance which dispatches change events, causing SetupPlot to update.

### Data Models

Our model of an optical setup, named Setup, is composed of a Detector, a Dye, an Excitation, and two Arrays of Filters, one for the excitation path, and another for the emission path. These four classes model the basic components of an optical setup and are composed of Spectrum properties: Detectors have qe, Dyes have absorption and emission, Excitation (sources) have intensity, and Filters have transmission and reflection. The Arrays of Filters are modelled with FilterStack, an Array-like class for Filters and their configuration, which has a Spectrum property for its total transmission. The Spectrum instances, used to represent the spectrum data for all components, are immutable objects, meaning that results from efficiency and transmission calculations can be efficiently cached in a WeakMap.

Collections of the different individual component types are modelled as DataCollection instances, a Map-like class which provides asynchronous and lazy access to the actual data. As with Setup instances, Collection instances dispatch events when a change happens, such as the addition or removal of elements, triggering an update of their corresponding Views and hence the user interface. Finally, a Setup-like class named SetupDescription is used to represent a Setup without actually creating instances of the required components. In a SetupDescription instance, the individual components are replaced by the component unique id which is used as a key on the respective DataCollection.

### Computation of efficiencies and brightness

The transmitted spectrum (‘mCherry (transmitted)’ in the plot legend of Figure 1) is the product of the dye emission spectra, all filters in the emission path, and the wavelength-dependent detector efficiency. The emission efficiency (‘em’ in the plot title of Figure 1) is the area of the transmitted spectrum, divided by the area of the dye emission spectrum.

The excitation efficiency (‘ex’ in the plot title of Figure 1) is the fraction of light from the excitation source absorbed by the dye. It is determined by evaluating the area of the source spectrum after modulation by all filters in the excitation path and the dye absorption spectrum, then dividing this area by the total area of the unmodulated excitation source spectrum. Because different spectra may have different ranges and resolutions, spectral values are interpolated at a resolution of 1 nm and assumed to be zero at all wavelengths outside the range covered by the provided data.

Relative brightness (‘brightness’ in the plot title of Figure 1) is approximated by the product of excitation efficiency, emission efficiency, dye extinction coefficient, and dye quantum yield. The final brightness is relative to 100% excitation and emission of Alexa-488 (Extinction Coefficient of 73000 M ^-1^ cm ^-1^, and Quantum Yield of 0.92), and multiplied by 10.

### SPEKcheck data source and format

In addition to the Javascript library and application, SPEKcheck includes a collection of spectra data for different dyes, excitation sources, filters, and detectors.

Data for most filters were found on their manufacturers’ web sites. The spectra for custom or unusual filters were requested from the manufacturer or, if unavailable, measured using a suitable spectrometer and light source. Data for dye extinction coefficients and quantum yields was retrieved from the suppliers’ web sites, the literature, or other web-based resources, such as http://www.fluorophores.org/. Spectra for lasers were modelled as unit pulses in wavelength with a 1 nm width, whereas those for polychromatic sources were measured with a spectrometer (CCS100, Thorlabs).

The files with the spectral data are plain text files containing a multi-line header with key/value pairs and comments, followed by a comma separated variable (CSV) section with the actual spectral values. The details of the format are included in the SPEKcheck documentation.

### Graphical User Interface

The SPEKcheck application reads from a template HTML file and injects it into a specified page element. This element may be the document body, so that SPEKcheck runs as a standalone, single-page application. Alternatively, other pages can import the same template and display it anywhere in the document, even allowing multiple instances of SPEKcheck in the same web page.

### External dependencies

SPEKcheck uses several external libraries. The javascript library Chart.js is used to handle plotting of the spectra. Bootstrap handles the front-end. jQuery is used by SPEKcheck to perform the asynchronous requests for the data files, and internally by Bootstrap to handle its events. All these libraries are free software under the MIT license, and compatible with GPL version which SPEKcheck is released under.

## Use cases

We have designed the SPEKcheck interface with a range of microscope users in mind:

1. Those with a specific microscope attempting to pick the ideal dye for their sample;
2. Those with access to multiple systems but an already prepared sample;
3. Those wishing to see how changes to individual filters and dichroics will change their detection efficiencies and image brightness.

In the first use case, having chosen a setup, the user presses the ‘Optimise Dye’ button, which estimates the efficiency of all available dyes for that setup.

In the second use case, after a dye has been selected manually, that selection will not be modified as different setups are selected. This allows a user to explore the efficiencies of different setups for a given dye.

In the third use case, a user may press the ’Customise Filters’ button to enable addition, removal, or modification of any elements in a setup. With no setup selected, this enables the user to build a completely new one from scratch. If a setup is selected, the user may assess the effects of making changes to that setup.

In all cases, SPEKcheck needs to be configured for the locally-available microscope systems and components, such as dichroics and detectors. While we host a public instance of SPEKcheck, we expect facilities to provide access to their own instances, configured for their own users and equipment. In this way, their users are not faced with long lists of filters, dyes, and other settings which are not appropriate to the equipment they have access to, and which could otherwise make searching for relevant settings an arduous task. The SPEKcheck library has been designed with this in mind, and may be used in any of three different ways: as an online single-page application, as a component of another web page, or as a local application that happens to run in a web browser.

### SPEKcheck as a dedicated single-page application

To customise SPEKcheck for their own users, facilities will need to define the microscopes they have available in the data/setups.json file, and ensure they have the data files for the relevant components. This is how we run our main SPEKcheck instance at Micron Oxford.

While this setup requires the facility to have access to a web server, SPEKcheck then runs completely on the client-side, simplifying the server setup and removing additional security implications.

### SPEKcheck as a component of another web page

The SPEKcheck application can be inserted into any HTML element and so does not need an entire page to itself, it will work as part of a larger web page. For example, a microscope facility can have individual web pages describing each of their microscopes, and have SPEKcheck embedded in those pages with only the related setups available for selection. Details on how to embed SPEKcheck on another page are included in the SPEKcheck documentation. It should be noted that SPEKcheck uses Bootstrap for styling, so the parent page style must not clash with the Bootstrap stylesheet.

### SPEKcheck as a local application

Since SPEKcheck is fully client-side, it can run offline and to support this, SPEKcheck releases include all dependencies. This enables a facility to make SPEKcheck available to their users on the same computer that they use to control a microscope, even if that computer has no access to the internet. It also enables individual users to configure a copy of SPEKcheck on their own computer for precisely the dyes and filters they require, without the need to run a web server. Facilities may also prepare their own releases of SPEKcheck, pre-configured with their setups, and distribute them to their users.

The Chrome and Safari web browsers will not, by default, allow file access from local applications and thus require a local web server or special configuration which has potential security implications. We recommend a local web server and provide instructions on one way to do this in the SPEKcheck documentation.

### Configuration

An instance of SPEKcheck requires a file describing pre-defined microscope setups, and data files containing the spectra for components used in those setups. If a site runs multiple instances of SPEKcheck, the setups for each instance can be defined in javascript code on the page itself, and each instance may have its own set of filters, detectors, dyes, and excitation sources.

The URL used to access SPEKcheck can include query elements that filter the available setups, filters, and dyes by a simple string match. Fragment identifiers may also be used to pre-select a microscope setup and a dye. This enables the creation of links for a specific setup or subset of available setups that can be bookmarked and shared. For example, at Micron, we run one instance of SPEKcheck on our web server that is configured with all our available microscope setups and data files; on each microscope computer, we provide a link from the desktop that opens SPEKcheck, displaying only the relevant setups for that microscope.

### JavaScript Library

As a JavaScript library we hope that SPEKcheck can also be used by other projects. First, the models and views are decoupled so the models could be used on other applications handling spectral data or fluorescence light path models. Second, the HTML code is inserted into the page and so could be modified to use other front-end libraries.

## Discussion

SPEKcheck uses widely available or easily obtainable data for light sources, dyes, filters, dichroics, and detectors to realistically model the fluorescence light path of fluorescence microscopes. Its significant advantage over other existing tools is that it models the propagation of both the excitation light to a sample and the fluorescence emission from the sample all the way to the detector. Additionally, it is able to use standard literature extinction coefficients and quantum efficiencies to estimate the absolute brightness of the detected signal, allowing comparison not only between different systems but also between different dyes on different systems. We have specifically designed SPEKcheck to be able to model complex fluorescence setups with an arbitrary number of dichroics and filters in both the illumination and emission path. Many modern advanced microscopes have such requirements. For instance, having a dual-view device to split two fluorescent channels across one camera requires two dichroics: one in the microscope filter cube and a second just before the camera. In these cases the spectral graphs can rapidly become cluttered and confusing. To mitigate this, individual traces can be toggled off and on by clicking on their entry in the legend. This enables clear visualisation of each component even in complex setups.

## Limitations of the SPEKcheck application

We made some conscious concessions in SPEKcheck with the aim of providing a simpler interface for the typical fluorescence microscope. These are:

- only one dye and detector can be selected at a time;
- filters in the excitation path are not displayed;
- the display range is limited to between 300 nm and 800 nm.

The SPEKcheck interface and the Setup class only allow the selection of a single dye. The main use of a configuration with multiple dyes would be to investigate bleedthrough which can still be accomplished. For example, in the case of a red/green configuration, first select the setup for the green channel, setting excitation and emission filters and light source, and select the green dye. Note its brightness. Then, keeping the setup the same, select the red dye. This will likely produce no brightness, so no red signal bleeds through into the green channel. Next, select the setup for the red channel and the red dye, and note its brightness. Then, keeping the setup for the red channel, select the green dye, and note its brightness. The relative values of these brightnesses is the relative intensity of signal from equal amounts of each dye in the red channel, and a good estimate of the expected bleedthrough.

Similarly, SPEKcheck has no support for dual or multi channel imaging. These are catered for by creating multiple setups with the same dichroic but different light sources, excitation filters, and emission filters.

Since multiple dyes and detectors can be modelled as multiple Setups, the SPEKcheck library can still be used to provide alternative interfaces in such cases. The Setup and SetupPlot classes are not singletons, and all the individual components are immutable objects, so it is possible to create a new interface that displays multiple setups at the same time. This would require a modified HTML template, and modifications to the presenter class to keep the multiple Setups in sync where appropriate.

In order to reduce the visual noise in the spectral plot, the filters in the excitation path are not displayed. Instead, the excitation light source is displayed after being attenuated by the excitation path filters and dichroics.

Finally, only the range of 300 nm and 800 nm is displayed in the plot preventing its use for two-photon imaging or similar. However, computations are done using the entire range of the spectrum data, even if they are not displayed in the plot. We have tested automatically adjusting the displayed range based on the spectra but this made the X axis change with each change of the setup, which prevented easy and direct visual comparison of resultant spectra. We selected the hardcoded range of 300 nm to 800 nm since it is a reasonable range for most fluorescence microscopy.

## SPEKcheck assumptions for computations

There are several fundamental assumptions in the calculations that should be considered when interpreting the numerical and spectral data from SPEKcheck produces.

1. Filters and dichroics can be used in either transmission or reflection. The underlying data can also be provided in either transmission or reflection. SPEKcheck transforms between these two options by assuming that the optics are lossless and that *reflection* = (1 *– transmission*).
2. Dye excitation is assumed to be the relevant region of the dye absorption spectra multiplied by the excitation light spectra after excitation filters and dichroics have been accounted for. This is likely true near the peak absorption of the dye. At wavelengths far from the peak, other transition states may be populated, so the model may overestimate the excitation efficiency.
3. The dye brightness is determined by multiplying by the extinction coefficient and the quantum yield of the dye. Extinction coefficients are usually measured at peak absorption so, again, the model may overestimate excitation efficiency at wavelengths far from this peak. In addition, the quantum yield is often extremely dependant on local environment. We make no account of either reduced extinction coefficient or changes to dye quantum yields.
4. We make no effort to account for illumination intensity or objective collection efficiency, usually dominated by the objective numerical aperture (NA). Our model should reflect true brightness changes across different excitation sources, dyes, and setups but only if the same illumination intensity is used and the same collected light spread over the same detector region. Moving from a 10× 0.3 NA objective to a 60× 1.4 NA objective will have a huge effect on the results.
5. SPEKcheck takes no account of dye photostability, which can significantly affect dye choice. For instance FITC will frequently be returned as the brightest dye in setups designed to detect green fluorescence. However, more modern dyes such as Alexa-488 or Atto-488 are almost always a better choice due to their substantially better photostability.
6. Imaging is often fundamentally signal to noise limited. Although we model the relative effect of different dyes, microscope setups, and detectors, we make no effort to determine true detectability. The brightness score should reflect the true relative brightness across setups, but factors such as detector noise levels or illumination intensity may mean that the specific signal in question is not detectable.

We note these specific limitations to ensure users are aware of the details behind the calculations SPEKcheck performs. The assumptions used enable us to generate realistic estimates of efficiencies and brightness using readily available data on dye, filters, dichroics, and detectors.

## Summary

SPEKcheck enables modelling of both simple and complex fluorescence light paths. It replaces error-prone and tedious spreadsheet-based methods for assessing the efficiencies of dye/filter combinations, optionally including the effects of specific light sources and detectors. We have found it extremely useful in helping users of our microscope facility choose the best dyes to use with our microscopes. It has also helped us choose replacement filters and dichroics to improve imaging with specific microscopes in biological systems that afford less flexibility in choice of dye.

We expect that it will be of use to others in the community of imaging facilities and their users, and have therefore released the full source code under the GNU GPL, along with spectral data for commonly used light sources, dyes, filters, dichroics, and detectors.

## Data availability

All data underlying the results are available as part of the article and no additional source data are required.

## Software availability

**Source code available from:** https://github.com/MicronOxford/SpekCheck/releases. **Archived source code at time of publication:** https://doi.org/10.5281/zenodo.1308103 ^7^.

**Licence:** GNU General Public License 3.0.
