# Supplementary material for: SPEKcheck — fluorescence microscopy spectral visualisation and optimisation: a web application, javascript library, and data resource
Source: Wellcome Open Res. 2018 Jul 30;3:92. [Version 1] doi: 10.12688/wellcomeopenres.14703.1 (PMC6107975; doi:10.12688/wellcomeopenres.14703.1)
Supplement: Peer review history

# Peer review: Referee response for version 1

**DOI:** 10.21956/wellcomeopenres.16012.r33602

**Published:** 2018-08-24

**Authors:** Alex Sossick

**Affiliations:** 1 Gurdon Institute, University of Cambridge, Cambridge, UK

SPECKcheck is an incredibly useful tool that has been developed to implement and understand the properties of fluorophores on a given microscope, which is incredibly useful especially for microscopy facilities where there are often numerous different types of microscopes.

 There are a number of complimentary/similar resources available, but the ability to uniquely compare fluorophores with an understanding based on the imaging system being used, should give researchers a useful input into experiment design. For example, being able to balance fluorophores, or understanding why on different microscope systems fluorophores have varying intensity ratios.

 SPECKcheck ran locally after downloading which was very elegant, a key to hosting local copies on specific microscopes. Especially in microscopy facilities with multiple systems, having a local copy of SPEKcheck specific to the system gives useful information to the microscope user of exactly what to expect from their fluorophore labelling on that system.

 Using relative brightness to Alexa-488 as a benchmark is astute, there are a number of options to reference against, but against a dye that is readily available, in common use and the characteristics in actual samples are understood makes a lot of sense.

 The display range of 300nm to 800nm is the standard range that the vast majority of users work within. As a future request it would be useful to be able to adjust this, for example it is rare for fluorophore emissions below 400nm to be imaged, so being able to personalise and select for example 400-700 would be a nicety, but not essential.

 There is further scope to develop SPECKcheck into a hosted resource for other microscopy facilities, or at least host standard microscope configuration files that can be loaded onto local instances.

I have read this submission. I believe that I have an appropriate level of expertise to confirm that it is of an acceptable scientific standard.

# Peer review: Referee response for version 1

**DOI:** 10.21956/wellcomeopenres.16012.r33600

**Published:** 2018-08-20

**Authors:** Teng-Leong Chew

**Affiliations:** 1 Advanced Imaging Center (AIC), Janelia Research Campus, Howard Hughes Medical Institute (HHMI), Ashburn, VA, USA

The software, SPEKcheck, created by Phillips, *et al.* and described in this manuscript could be a very useful tool for imaging core users and personnel to help determine optimum setup of their imaging experiment. Considering this is an open source platform with some customization (e.g. importation of particular filter parameters), it allows for great flexibility for core staff to design SPEKcheck profiles particular to the instruments in their facility. Below are a few suggested revisions and/or additions that would broaden the applicability of the software to users and imaging specialists outside of core facilities.

  

1. The most significant omission from the software is the ability to calculate the emission of multiple dyes in a single experiment. The authors state in the discussion the “main use of a configuration with multiple dyes would be to investigate bleedthrough…”; I do not agree with the assumptions inherent in this statement. Primarily, the authors assume that the user will never have an instance of using a single emission filter in a multi-color experiment. In experiments that require multiple fluorescent labels but only a single mutli-bandpass emission filter, a tool like SPEKcheck would be invaluable. Here, bleedthrough, while still a concern, is not the primary issue; it is more important to know that the correct multi-band pass filter was selected to minimize bleedthrough AND optimize the emission windows. As you begin to add more fluors, noting the brightness of each individual fluor and tallying that up is veering towards the spreadsheet method, which the authors state SPEKcheck was designed to avoid. Furthermore, allowing the addition of fluorophores to the spectrum ad hoc (similar to how the software already handles multiple filters) would broaden the potential audience to users of more custom-built microscopes that still require optimization of fluorophore, laser, and filter selection.
2. It would be more accurate if the software updated the amount of emitted light depending on the laser or white light chosen (i.e. the excitation efficiency based on absorbance). Currently, the software calculates the emission based on the efficiency of the camera, the spectra of the fluorophore, and the filters used. However knowing whether you are optimally exciting the chosen fluorophore (beyond just the percentage given) is of critical importance. It would also be helpful to know in multicolor experiments how much cross-excitation may occur, given the excitation source and excitation filters in place.

I have read this submission. I believe that I have an appropriate level of expertise to confirm that it is of an acceptable scientific standard.

# Peer review: Referee response for version 1

**DOI:** 10.21956/wellcomeopenres.16012.r33601

**Published:** 2018-08-09

**Authors:** Michelle Peckham, Ruth Hughes, Alistair Curd

**Affiliations:** 1 Astbury Centrefor Structural Molecular Biology, School of Molecular and Cellular Biology, Faculty of Biological Sciences, University of Leeds, Leeds, UK; 2 Astbury Centre for Structural Molecular Biology, School of Molecular and Cellular Biology, Faculty of Biological Sciences, University of Leeds, Leeds, UK

- Overall, the paper has a helpful introduction to the current ability to determine the optimal microscope set up and dye choices and the advantages of the new SPEKcheck interface. Acknowledgement of its limitations is helpful in knowing where its fits within the resources already available. As the paper emphasises this would be useful for those working in imaging facilities and the paper gives good details and instructions about implementing for specific microscope setups and website configurations.
- The information produced by SPEKcheck has a number of caveats, making it useful as a check, to give a general rule of thumb but perhaps not helpful for more complex questions.

- Without attempting to install and use it for our facility, it sounds useful. It is likely to be useful to others, through its ability to add relevant components and remove irrelevant components.
- A further advantage is this is a good web-based approach, which means it is easily accessible, and does not to have to re-invented for every facility/microscope development lab. This is mainly about convenience, because to add the components that are relevant to any particular application, the user would need to get hold of the performance vs. wavelength data anyway, and an experienced microscopist could then perform the multiplication of the components' properties, to get total performance. However, to see several results quickly as you can do here would take some more coding, and less experienced microscopists, and particularly those without a strong background in optics will find this useful.The wider impact is less clear; in that it’s uncertain if users of microscopes in a facility who might need to make choices about the optimal set up for the microscope and dye they are using, would quickly be able to get to grips with the software, and know which settings to include. However, the software could be helpful in enabling discussions with facility staff before imaging to help people plan their experimental setup or as a helpful training tool.
  Specific comments are:

1. It would be good to see an example of how the truncation of the display at 800 nm does not affect the modelling and calculation results - and how to use the software in that case. Two-photon is mentioned for instance, and there are dyes/other fluorescent chemicals that go beyond this wavelength and below 300 nm.
2. There don't seem to be too many references? This is quite a niche paper but I thought there would be more papers that would be relevant to cite.
3. A little bit more explanation on the website itself would be useful for those with less optical experience, in order to make this more broadly applicable.  I think this is particularly important, because in the future, most users are likely to go straight for the website, without necessarily reading the paper first.  It will be important to maintain and keep the website going, and provide as much explanation as possible.  A ‘wiki’ approach might also be useful here.
4. Some issues we spotted were: for Zeiss, which it is possible to change the set up (type of microscope), dye, excitation and detector, the band pass filter can’t be changed.The list of cameras provided for Olympus TIRFM is somewhat limited, there doesn’t appear to be an EMCCD camera? And the cMOS cameras listed do not include the 95B camera. Why are Zeiss GAASP and PMT still in selection list for the Olympus TIRFM (and others) when it’s unlikely that they will be there on the system?  For PALM – what is the default detector (just indicated by a ‘tick’)?
5. Using the Safari web browser, we couldn’t immediately spot the symbol in the top right (three lines) – to change filters etc in the set up – this could be indicated more helpfully.
6. Sadly, we were able to delete both filters in the excitation path, and then it wasn’t immediately clear how to add anything back! It was only on re-reading the paper that we discovered that filters could be dragged back in. 
7. It would be helpful to state what T and R refer to in the excitation and emission path on this page, and clearly indicate that there is the possibility to ‘click to drag into Emission or Excitation path (for the Filters).

We have read this submission. We believe that we have an appropriate level of expertise to confirm that it is of an acceptable scientific standard.
